# Opioid-free anesthesia for a child with trisomy 13 with obstructive sleep apnea: a case report

**DOI:** 10.1186/s40981-020-00354-3

**Published:** 2020-06-11

**Authors:** Makiko Yamamoto, Izumi Miyazaki, Hiroaki Kishikawa, Atsuhiro Sakamoto

**Affiliations:** Department of Anesthesiology, Nippon Medical School, 1-1-5 Sendagi, Bunkyo-ku, Tokyo, 113-8602 Japan

**Keywords:** Central apnea, General anesthesia, Non-narcotic, Obstructive sleep apnea, Opioid-induced respiratory depression, Opioid-free, Tonsillectomy, Trisomy 13

## Abstract

**Background:**

Most children with trisomy 13 display central apnea, and are prone to opioid-induced respiratory depression. We conducted opioid-free anesthesia for a patient with trisomy 13 and obstructive sleep apnea, and safely extubated the patient in the operating room.

**Case presentation:**

A 27-month-old girl with trisomy 13 underwent tonsillectomy. Given her high sensitivity to opioids, general anesthesia was introduced and maintained only with 2–5% sevoflurane and 33% nitrous oxide in oxygen. We used acetaminophen for postoperative analgesia. The tracheal tube was removed under stable breathing pattern 10 min after the surgery in the operating room. Two years later, opioid-free anesthesia with 2–5% sevoflurane and 33% nitrous oxide in oxygen was again performed safely for tube insertion into both eardrums.

**Conclusion:**

Opioid-free anesthesia with adequate non-narcotic analgesics is safe for children with trisomy 13 with multiple apnea-related comorbidities.

## Background

Trisomy 13 is a chromosomal disorder resulting in an anatomically abnormal airway, central nervous system abnormalities that include respiratory depression, and congenital heart disease [1]. The incidence of this pathology is approximately 1 per 10,000 births, with a neonatal mortality rate of 75%, and a 1-year survival rate after birth as low as 10% [2–4]. Death is reportedly most often caused by lethal apnea, rather than heart failure [5].

Anatomically abnormal airways, such as cleft lip and palate, other facial deformities, laryngomalacia, and tracheobronchomalacia, can cause obstructive sleep apnea (OSA) [6]. On the other hand, the severity of central apnea is reportedly an important factor related to refractory apnea or lethal apnea [7, 8].

While opioids are included in postoperative analgesia for many surgeries requiring general anesthesia, opioid-free anesthesia can be performed to prevent adverse effects including opioid-induced respiratory depression. Risk predictors for opioid-induced respiratory depression include the 24-h period after surgery, airway surgeries, OSA, and developmental delay [9]. In such cases, non-narcotic analgesics may be a better option.

Because children with trisomy 13 have a frail central nervous system, they show heightened sensitivity to opioids. General anesthetic management for these individuals needs strategic consideration, especially for stabilization of the respiratory condition after the tracheal tube is extubated. Opioid-free anesthesia is one such strategy. A case of tonsillectomy for a girl with trisomy 13 with OSA resulting in chronic hypoxemia is reported. This case report is structured according to the CARE guideline [10].

## Case presentation

A 27-month-old girl with trisomy 13 presented with OSA. She was born at 38 weeks’ gestational age and hospitalized because of low birth weight. She was deaf and had been diagnosed with mosaic-type trisomy 13. Tracheomalacia and central apnea required ventilation support, but tracheomalacia had resolved 3 months after the birth with her growth, and central apnea had shown gradual improvement. She had been discharged without respiratory support at 8 months old. OSA had appeared at 2 years old due to increasing adenoid hypertrophy. Her parents reported snoring and apnea at night. She was on the monitor of peripheral oxygen saturation (SpO_2_) at home, and home oxygen therapy started after identifying SpO_2_ ≤ 80% on room air. Tonsillectomy under general anesthesia was scheduled for OSA and repeated otitis media.

**Fig. 1:**
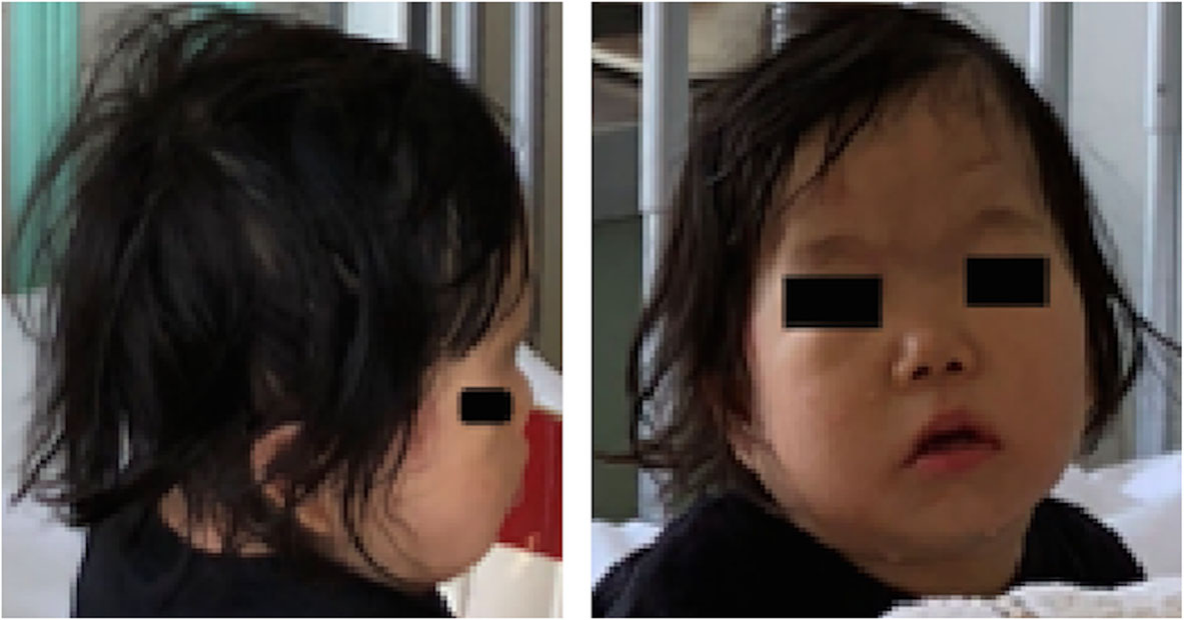
Appearance of the head and neck. Pictures were taken during this hospitalization

At the time of hospitalization for surgery, her height was 76 cm, and her weight was 10 kg. Growth and development were equivalent to a child of about 18 months old. Preoperative chest X-ray and electrocardiogram examinations were normal. She had a patent ductus arteriosus that had been followed-up until this hospitalization. The latest transthoracic cardiac ultrasonography showed that the shunt flow was left-to-right, with a maximum velocity of 4.30 m/s, a maximum pressure gradient of 74.0 mmHg, and a pulmonary/systemic blood flow ratio of 1.1. Microcephaly and retrognathia were apparent (Fig. 1). Intermittent central apnea was present as one of the central nervous system complications of trisomy 13.

We did not provide her premedication before the induction of general anesthesia. General anesthesia was induced with sevoflurane up to 5% and 33% nitrous oxide in oxygen [11]. OSA made mask ventilation difficult even though two anesthesiologists were involved in positioning the head, moving the mandible forward, and opening the mouth to facilitate ventilation. She was placed in a lateral position and administered 100% oxygen immediately after SpO_2_ began to decrease. Spontaneous respiration resumed in the lateral position, and SpO_2_ improved. She became apneic intermittently with 100% oxygen and 5% sevoflurane, but assisted mask ventilation was easy in the lateral position. Then, we inserted a peripheral venous line. We administered 0.01 mg/kg of atropine and 1 mg/kg of rocuronium intravenously. We performed tracheal intubation easily using a Macintosh-type laryngoscope. Two percent sevoflurane and 33% nitrous oxide in oxygen remained throughout the rest of the case. Vital signs remained stable during the procedure without opioids and benzodiazepines. We administered 10 mg/kg of acetaminophen suppository for postoperative analgesia.

**Fig. 2:**
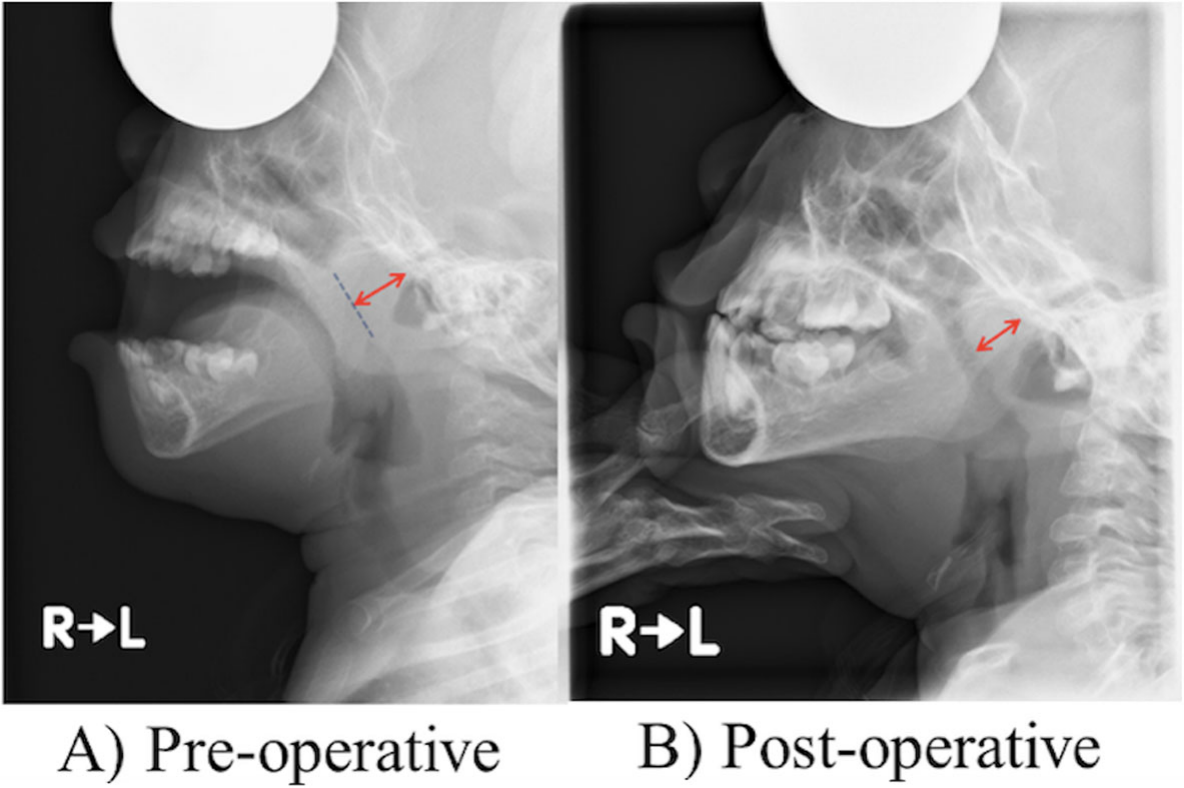
Pre- and postoperative lateral-view radiographs of the nasopharynx. **a** Preoperative image. **b** Postoperative image. Bloated adenoids (**a**: arrow) encroach on and severely obstruct the nasopharyngeal airway (**a**: dotted line). After tonsillectomy (**b**: arrow), the nasopharyngeal airway is open, allowing the patient to breathe with the mouth closed

The operation took 36 min, and spontaneous breathing resumed 10 min after discounting inhalational anesthetics. We administered 4 mg/kg of sugammadex sodium before extubation in the operating room. Her respiratory condition was monitored postoperatively in a high-care room with a stand-by ventilator for the rest of the day. It was because congenital central apnea due to trisomy 13 is prone to happen even with inhalational anesthetics. No apnea or desaturation was apparent. She was transferred to the general pediatric ward on postoperative day 2 and went home on postoperative day 4. Radiographs confirmed that the nasopharyngeal airway was open following the tonsillectomy (Fig. 2). Home nasal oxygen was no longer needed postoperatively.

Two years later, we reapplied the same anesthetic protocol successfully during tube insertion into both eardrums for repeated otitis media. At this time, she had grown to 88 cm in height, and weight had increased to 13 kg. Growth and development were equivalent to a child of about 30 months old. We did not provide her premedication. General anesthesia was introduced and maintained only with 2–5% sevoflurane and 33% nitrous oxide in oxygen. We initially tried to maintain her spontaneous breathing effort during surgery. However, we noticed mild OSA and intermittent apnea. Therefore, we intubated the patient and controlled her ventilation, given the previous experience of easy tracheal intubation. The operation took 20 min, and we extubated the patient under a stable breathing pattern in the operating room. The patient was fully awake and was, therefore, moved directly to a general pediatric ward with SpO_2_ monitor only at that time. No postoperative analgesics were needed.

## Discussion

Opioid-free anesthesia was safely performed for tonsillectomy in a child with trisomy 13 who presented with OSA, which could cause opioid-induced respiratory depression. Adequate postoperative pain relief was achieved with an acetaminophen suppository. This strategy made the respiratory condition stable, allowed early withdrawal of ventilator support after the operation, and was reproducible.

Opioid-free anesthesia is thought to prevent postoperative central apnea resulting in delayed extubation or re-intubation of a tracheal tube. Naloxone events caused by opioid-induced respiratory depression have been classified into six types, and precautions for each have been considered [9]. A reduction in opioid dose of at least 50% has been suggested for children with significant airway and apnea-related comorbidities, according to the reported classification [9]. This theory was applicable in the present case because of central apnea caused by trisomy 13.

OSA is also a special health condition requiring opioid dose reduction. Previous work has highlighted quantitative changes in opioid receptors in the brainstem that lead to increased sensitivity to opioids or benzodiazepines in children with chronic hypoxemia [12]. An anesthetic regimen for tonsillectomy in children has been presented, in which adjustments were made to the doses of midazolam and opioid depending on the severity of OSA [13]. Mask induction has been reported as safe with 30% nitrous oxide and sevoflurane up to 8% in oxygen, regardless of the severity of OSA. The patient in our case presented with OSA, which made mask ventilation difficult, but this problem was resolved by placing the patient in a lateral position. An oral airway could have been effective. Although administration of a muscle relaxant was avoided in the reported regimen, rocuronium was safely reversed with an adequate dose of sugammadex sodium.

The patient in this case presented with one of the most severe chromosomal triplication diseases with central apnea, in addition to OSA, with SpO_2_ reduced to approximately 80% in room air. The risk in the present case could have been higher than in other cases referred to when considering the prescribed strategies for opioid-induced respiratory depression [9]. Opioid analgesics needed to be replaced by non-narcotic analgesics as much as possible and the respiratory condition needed postoperative monitoring in the present case [9]. Opioid-free anesthetic management that focused on safe early extubation in the operating room for a child with trisomy 13 was reported for the first time.

One limitation in the present case was the difficulty in accurately evaluating analgesia. The patient showed mental delay and was deaf. Insufficient pain relief may have caused unstable breathing, laryngeal spasm, or dangerous excitement. For sufficient multimodal analgesia, local anesthesia could have been added. Another limitation was that nitrous oxide should be avoided in some cases, including those with ileus, increased intraocular pressure, increased tympanic pressure, or brain surgery. Otitis media may have excessively increased tympanic pressure if the anesthetic time had been longer. Finally, remifentanil can be used for tracheal intubation and maintenance of general anesthesia, since remifentanil has a short context-sensitive half-time of a few minutes.

## Conclusion

Opioid-free anesthesia is appropriate for patients who have high sensitivity to opioids. Opioid-free anesthesia that provides sufficient pain relief can be performed with multimodal analgesics in children with trisomy 13 and OSA, which causes chronic hypoxemia, or any other apnea-related comorbidities.

## Data Availability

Data sharing is not applicable to this article as no datasets were generated or analyzed during the present study.

## Abbreviations

OSA: Obstructive sleep apnea
SpO_2_: Peripheral artery oxygen saturation
